# Devitalizing the Dental Pulp, and Treatment Preparatory to Filling

**Published:** 1869-05

**Authors:** J. P. Wilson


### ARTICLE VIII.

### *Devitalizing the Dental Pulp, and Treatment Preparatory to Filling.*

By Dr. I. P. Wilson.

When teeth are so badly decayed as not to admit of a chance of saving the pulp alive, I have practiced the following treatment with gratifying results.

And in writing on this subject, I shall particularize for the benefit of those who have no regular method of treating such teeth, and consequently have poor success, if not entire failure:

Before treatment I remove but little disease from the cavity—perhaps only syringe it out with warm water. If it is an approximal and the decay extends below the margin of the gum, it is important that an application of sandarac varnish be made to that portion of the gum exposed to the cavity, that the absorbents may be closed. If this precaution is not observed, the arsenious acid will act upon the surrounding parts, pericementitis will follow, and failure is a probable result.

This being done I take a small pellet of cotton, moisten it with creosote, touch one side of this to arsenic, (either dry or in paste) and apply directly over the pulp.

Another pellet of cotton saturated with sandarac varnish should be in readiness, and applied over the first, filling the cavity full. This will harden, and prevent the arsenious acid from escaping into the mouth and doing injury.

I now dismiss my patient with directions to call again in just 24 hours. At the expiration of this time I remove the sandarac plug, and almost invariably find the pulp dead, so that it can be removed with little or no pain.

At this sitting I generally prepare the cavity for filling, by removing not only the bulk of pulp, but the nerve vessels in the dental canals, so far as it is practicable to do so; and in order to do this, I sometimes find it necessary to enlarge one-third or one-half the length of the canal with a bur drill, that I may gain access to the root vessels, and thereby make the operation much more thorough, and I believe productive of better results.

But we frequently find the nerve vessels alive, and quite sensitive, but the pain is only momentary, and generally ceases as soon as the instrument is withdrawn.

In such cases I have been in the habit of entirely extirpating these vessels if possible, believing if this is not done, they will afterwards perish, and become foreign matter. This may be done with a broach, or by making a second application with the arsenic. The former I consider a much better way, and always practice it when my patient will allow me to do so.

In using arsenic a second time, it circulates more generally throughout the entire structure of the tooth, reaching perhaps the nutritive vessels of the periosteum, and thereby cutting off all nourishment to the tooth, and it becomes necrosed. I do not believe that a second application of arsenic will produce such results as this as a rule, but it is *liable* to do so, and therefore barely enough should be used to accomplish the object, and *no more.*

The cavity being prepared, I rinse it with warm water, dry it thoroughly, and then force creosote into the canals on very small pieces of cotton, or thread is better, as one end of it can be left in the outer cavity, by which it can be removed very readily.

This being done I request my patient to call again in a week or ten days. I then remove the temporary filling, and ascertain the condition of the tooth.

If it should be elongated, and concussion produces pain, I would not plug, but commence treatment for pericementitis. And if the small piece of cotton or thread in the root canals should produce a fetid odor, and indicate an unhealthy condition, I would not think of filling, but would rinse out the canals thoroughly with warm water, and again apply the creosote, and so continue to do from time to time, until a normal condition is present. But if on removing the temporary filling, I find the tooth in a good condition, I proceed at once to fill the canals ( if I have gained access to them), and then the main cavity.

In cases of this kind where an expensive gold filling is required, I think it best as a general rule to put in a *test* plug at first, and then if all is well, fill it with gold from two to to four months subsequently.

But a word in regard to these *test fillings:* When the oxychloride of zinc is used for this purpose, it is simply *no test at all*, as it is very porus, and the gases can easily escape through it. I would therefore prefer Hill-stopping for this purpose, or at least I would close up the openings into the canals with the Hill-stopping, and then the oxychloride may be used for the balance of the filling if it is preferred.

This has been my method of treating such teeth, and I have very seldom had any further trouble with them.

I do not know that I am offering anything new on this subject, but I trust the above suggestions may serve as a guide to those who have heretofore been unsuccessful in the treatment of such teeth.—*Missouri Dental Journal.*

---

